# Adolescents’ and Young Adults’ Receipt of Person-Centered Contraceptive Counseling

**DOI:** 10.1001/jamanetworkopen.2025.51287

**Published:** 2025-12-26

**Authors:** Brooke Whitfield, Tracey A. Wilkinson, Laura D. Lindberg

**Affiliations:** 1 Reproductive Equity Action Lab (REAL), University of Wisconsin School of Medicine and Public Health, Madison; 2 Division of Children’s Health Services Research, Department of Pediatrics, Indiana University School of Medicine, Indianapolis; 3 Department of Urban-Global Public Health, Rutgers School of Public Health, Newark, New Jersey

## Abstract

**Importance:**

Access to person-centered contraceptive counseling (PCCC) is critical for adolescents and young adults. There is limited data on the quality of contraceptive counseling that young people are receiving in the US

**Objective:**

To assess the quality of contraceptive counseling received by adolescents and the association with use of a preferred method of contraception.

**Design, Setting, and Participants:**

This survey study used US population-based data from the 2022 to 2023 National Survey of Family Growth (NSFG). Participants included self-identified female respondents who had a contraceptive care visit in the past year and responded to the PCCC items. The analytic sample included 261 adolescent respondents aged 15 to 19 years, 276 young adult respondents aged 20 to 24 years, and 1506 adult respondents aged 25 years or older. Data were analyzed from December 2024 to July 2025.

**Main Outcomes and Measures:**

Receipt of PCCC and preferred contraceptive method use. Multivariable logistic regression analyses and 95% CIs were used to assess data.

**Results:**

This study analyzed data from 2043 self-identified female respondents. The sample was nationally representative, with 12.8% (95% CI, 10.2%-15.8%) of respondents identifying as Black, 19.5% (95% CI, 15.7%-23.9%) as Hispanic, and 58.6% (95% CI, 53.8%-63.3%) as White. Additionally, 57.6% (95% CI, 53.4%-61.8%) lived above 250% of the federal poverty line, and 66.1% (95% CI, 62.7%-69.2%) received birth control services from a private doctor's office or health maintenance organizations facility. Receipt of PCCC was low across all age groups. However, adolescents aged 15 to 19 years had significantly lower odds of receiving PCCC than adults aged 25 years or older (adjusted odds ratio [aOR], 0.61; 99% CI, 0.37-0.99). There were no differences in receipt of PCCC between young adults aged 20 to 24 years and adults aged 25 years and older (aOR, 0.83; 99% CI, 0.55-1.24). Adolescents who received PCCC had significantly higher odds of using their preferred contraceptive method than adolescents who did not receive PCCC (aOR, 2.35; 95% CI, 1.06-5.21). There were no differences in preferred contraceptive method use by PCCC among young adults (aOR, 0.57; 95% CI, 0.25-1.26) or adults (aOR, 1.01; 95% CI, 0.70-1.45).

**Conclusions and Relevance:**

In this survey study of self-identified female respondents, we found that adolescents were less likely than adults age 25 years or older to receive PCCC, but adolescents who did receive PCCC reported higher rates of preferred method use. These findings support ongoing efforts to improve the quality of contraceptive counseling for adolescents through person-centered approaches that provide comprehensive information, respect their preferences, and support their autonomy.

## Key Points

**Question:**

Are US adolescents and young adults receiving person-centered contraceptive counseling (PCCC), and is receipt associated with preferred method use?

**Findings:**

This survey study of 2043 female respondents used the 2022 to 2023 wave of the National Survey of Family Growth and found that receipt of PCCC was low across all age groups; however, adolescents (aged 15 to 19 years) were less likely to receive PCCC than adults aged 25 years and older. Receipt of PCCC was positively associated with preferred method use among adolescents, but not adults.

**Meaning:**

These findings suggest that two-thirds of adolescents did not receive PCCC, but those who did were more likely to use their preferred method; clinicians should prioritize PCCC for adolescents.

## Teaser

This survey study assesses the quality of contraceptive counseling received by adolescents and its association with use of a preferred method of contraception using data from the 2022 to 2023 National Survey of Family Growth.

## Introduction

Changes to the contraceptive care landscape in the past decade, such as the contraceptive coverage mandate in the Patient Protection and Affordable Care Act, Medicaid expansion, updated clinical practice guidance specific to adolescents, and adoption of clinical practices, such as insurance coverage for extended prescriptions, have greatly expanded contraceptive access for adolescents and young adults.^1,2,3,4,5,6,7^ Additionally, the growth of telehealth and online contraceptive services, as well as the expansion of prescribing pharmacists and over-the-counter contraception, have diversified pathways to contraceptive care access.^8,9,10,11,12,13^ Despite this tremendous progress, access to contraception is still highly inequitable and, oftentimes, restricted for adolescents and young adults.^14^

Furthermore, while contraceptive access for adolescents and young adults is critical for reproductive autonomy, it is not sufficient. Research shows that the quality of contraceptive counseling people receive is linked to their contraceptive use, satisfaction, and well-being.^15,16,17^ A key indicator of high-quality contraceptive care is the extent to which it is person-centered (also referred to as patient-centered).^16^ Person-centered contraceptive counseling (PCCC) focuses on respecting patients’ values and preferences and is considered a key aspect of high-quality family planning care.^16,18,19^ The PCCC scale is a validated 4-item measure of counseling quality and patient satisfaction.^20,21^ The PCCC is recommended by the US Office of Population Affairs for monitoring the provision of quality family planning.^22^

Initial inclusion of the PCCC scale in the 2017 to 2019 National Survey of Family Growth (NSFG), a key national survey of sexual and reproductive health, allowed for investigation of receipt of high-quality PCCC among reproductive-age women aged 15 to 49 years. Findings show that receipt of high-quality PCCC was low across all ages (approximately 50%) but were inconclusive in detecting whether adolescents and young adults experience different rates of PCCC than adults.^23^ One regional study in the US south found that minors (aged 16 to 17 years) were less likely to report receipt of PCCC than older adolescents and young adults (aged 18 to 24 years).^24^ Expanding and monitoring the receipt of PCCC for adolescents and young adults is essential, given the positive association between PCCC and preferred method use that has been identified in broader population studies.^15,25^ Lastly, given recent legal and policy shifts, it is increasingly vital to ensure that adolescents and young adults receive person-centered contraceptive counseling that is responsive to their unique needs.^26,27,28^

To address the current gaps in the literature, this study uses the most recent 2022 to 2023 wave of the NSFG to compare the receipt of PCCC among adolescents (aged 15 to 19 years), young adults (aged 20 to 24 years), and adults (aged 25 to 49 years), analyzing differences by demographics and contraceptive care factors. Additionally, to assess the role of PCCC in shaping chosen contraception, we examine associations between PCCC scores and the use of preferred contraceptive methods within age groups.

## Methods

The institutional review board (IRB) at the National Center for Health Statistics (NCHS) approved the NSFG data collection methods. Informed consent procedures were approved by the NCHS Ethics Review Board. Electronically signed parental permission and minor assent were obtained for all minor respondents. Adult respondents could provide their consent verbally without a signature. This study followed the American Association for Public Opinion Research (AAPOR) reporting guideline. Because our analysis used publicly available, deidentified data, the IRB at the University of Texas at Austin, the first author’s institution at the time of analysis and submission (B.W.), granted this study exempt status.

### Data

This analysis used data from the 2022 to 2023 NSFG, a nationally representative survey of women and men aged 15 to 49 years that collected information about family formation, contraception, and sexual health. The NSFG uses a multistage probability sampling design that oversamples Black and Hispanic participants and adolescents aged 15 to 19 years. Full details about the survey methods are available online.^29^ We limited these analyses to self-identified female respondents who reported receiving contraceptive counseling and/or a contraceptive method during a health care visit in the past year, and who therefore received the PCCC questions in the survey instrument.

### Measures

The primary outcomes of interest were the PCCC scale, and a binary measure of preferred contraceptive method use. The PCCC asked respondents to rate their most recent experience with their family planning clinician on a 5-point scale from poor to excellent on 4 items: (1) “How did this provider rate on respecting you as a person?”; (2) “How did this provider rate with respect to letting you say what mattered most to you about your birth control method?”; (3) “How did this provider rate on taking your preferences about birth control seriously?”; (4) “How did this provider rate on giving you enough information to make the best decision about your birth control method?” We reported distributions for each scale item and created a binary measure identifying respondents who answered excellent vs very good, good, fair, or poor. We also created a binary summary measure indicating whether a respondent answered excellent to all 4 questions, which has been identified as a benchmark for high-quality contraceptive counseling care by the PCCC scale creators, who psychometrically tested the measure.^20^ Preferred contraceptive method use was a binary measure based on responses to the following item: “If you did not have to worry about cost and could use any type of contraceptive method available, would you want to use a (different) method?” This was asked of all respondents, regardless of their current contraception use.

Covariates of interest included race and ethnicity (Hispanic, non-Hispanic Black, non-Hispanic other, and non-Hispanic White), English proficiency, household income level (less than or equal to 100% Federal Poverty Level [FPL] vs greater than 100% FPL), and where birth control services were received (private doctor's office or health maintenance organization [HMO] facility, public clinic, other) given their previously documented associations with contraceptive outcomes. Race and ethnicity categories were defined by the NSFG using the Office of Management and Budget classifications. Non-Hispanic other included American Indian or Alaska Native, Asian Indian, Chinese, Filipino, Japanese, Korean, Vietnamese, Other Asian, Native Hawaiian, Guamanian or Chamorro, Samoan, and Other Pacific Islander. English proficiency was coded as high for respondents who answered very well to the question “How well do you speak English?” and low for all other responses (not at all, not well, well). This cut point aligns with the US Census Bureau’s designation of limited English-speaking households.^30^ Public clinics include community health clinic, community clinic, public health clinic, family planning or Planned Parenthood clinic, school or school-based clinic, and hospital or outpatient clinic. Other included employer or company clinic, other hospital location including emergency room, urgent care center or walk-in facility, in-store health clinic, and some other place.^31^

We included sexual orientation (queer vs heterosexual), survey mode (face-to-face vs web mode), whether a respondent was a minor (aged 15 to 17 years), mother’s education (less than high school vs high school diploma vs some college or more), urbanicity (principal city of Metropolitan Statistical Area [MSA], other MSA, not MSA), ever had sex, number of years since sexual debut, whether the respondent had a child, and how they paid for family planning services (insurance vs Medicaid vs out-of-pocket vs other) in initial models based on their theoretical relevance to the outcomes of interest. We then used stepwise regression to identify the subset of covariates that provided the best model fit, listed above.

### Statistical Analysis

Differences in responses to individual PCCC items and the percentage who reported excellent on all 4 items across age groups were evaluated by examining overlap in 95% CIs. Then we estimated bivariate and multivariable logistic regression models to test for significant differences in the likelihood of reporting excellent on individual PCCC items and across all 4 items, by sociodemographic characteristics. To adjust for multiple comparisons, we applied the Bonferroni correction and evaluated significance by examining overlap in 99% CIs. To understand the association between receipt of PCCC and preferred contraceptive method use within age groups, we conducted multivariable logistic regression models and evaluated overlap in 95% CIs. All multivariable models control for race and ethnicity, English proficiency, income, and where birth control services were received.

To avoid centering Whiteness, we compared the likelihood of our outcomes of interest within a given subgroup with everyone not in that subgroup (eg, Black vs everyone who is not Black) instead of using an arbitrarily selected reference group, such as White race.^32^ All analyses were weighted using NSFG-designated survey weights and the *svy* command in Stata version 18.0 (StataCorp). Data were analyzed from December 2024 to July 2025. Statistical significance was set at *P* < .05, and all tests were 2-sided.

## Results

The final analytic sample of those who answered all 4 PCCC questions included 261 respondents aged 15 to 19 years, 276 respondents aged 20 to 24 years, and 1506 respondents aged 25 years or older. Of the total 2043 respondents, 12.8% (95% CI, 10.2%-15.8%) identified as Black, 19.5% (95% CI, 15.7%-23.9%) Hispanic, 9.2% (95% CI, 7.2%-11.6%) non-Hispanic other, and 58.6% (95% CI, 53.8%-63.3%) White (Table 1). Adolescents reported higher frequencies of living at or below the FPL (23.9%; 95% CI, 18.3%-30.6%) than adults (15.3%; 95% CI, 12.9%-18.3%). Adults reported higher frequencies of visiting a private doctor's office or HMO facility office for birth control services (71.1%; 95% CI, 67.2%-74.7%) than adolescents (52.8%; 95% CI, 44.3%-61.2%) and young adults (58.1%; 95% CI, 51.4%-64.6%). Sociodemographic characteristics of the full female-identifying NSFG sample are presented in eTable 1 in Supplement 1.

**Table 1.** Sample Characteristics, Female-Identifying Respondents Who Received a Birth Control Method and/or Counseling in the Past Year, National Survey of Family Growth, 2022-2023

Characteristic	Respondents, No. (weighted %) [95% CI]			
	Total (N = 2043)	15-19 y (n = 261)	20-24 y (n = 276)	≥25 y (n = 1506)
Race and ethnicity				
Black	295 (12.8) [10.2-15.8]	37 (10.6) [6.9-15.8]	45 (16.0) [11.1-22.5]	213 (12.3) [9.8-15.3]
Hispanic	411 (19.5) [15.7-23.9]	63 (19.7) [13.6-27.7]	66 (18.1) [13.1-24.4]	282 (19.8) [16.0-24.4]
White	1123 (58.6) [53.8-63.3]	135 (60.0) [51.9-67.7]	138 (57.6) [49.5-65.4]	850 (58.6) [53.4-63.6]
Other^a^	214 (9.2) [7.2-11.6]	26 (9.7) [6.2-14.8]	27 (8.3) [5.0-13.4]	161 (9.3) [6.9-12.4]
English proficiency				
Low	187 (9.2) [7.2-11.6]	20 (6.0) [3.4-10.4]	20 (6.6) [3.9-10.8]	147 (10.6) [8.2-13.5]
High	1849 (90.8) [88.4-92.8]	239 (94.0) [89.6-96.6]	256 (93.4) [89.2-96.1]	1354 (89.4) [86.6-91.8]
Income level, % FPL				
0-100	342 (16.8) [14.5-19.4]	69 (23.9) [18.3-30.6]	59 (16.5) [12.2-22.0]	214 (15.3) [12.8-18.3]
101-250	527 (25.6) [22.5-29.0]	70 (26.0) [19.5-33.7]	87 (31.4) [24.1-39.7]	370 (23.9) [20.8-27.3]
>250	1174 (57.6) [53.4-61.8]	122 (50.1) [42.1-58.0]	130 (52.1) [43.0-61.0]	922 (60.8) [56.6-64.7]
Where birth control services were received				
Private doctor's office or HMO facility	1359 (66.1) [62.7-69.2]	136 (52.8) [44.3-61.2]	158 (58.1) [51.4-64.6]	1065 (71.1) [67.2-74.7]
Public clinic	381 (19.1) [16.4-22.1]	64 (27.8) [20.4-36.6]	68 (22.7) [16.5-30.3]	249 (16.2) [13.5-19.5]
Other^b^	293 (14.9) [13.0-16.9]	60 (19.4) [14.3-25.8]	50 (19.2) [14.2-25.5]	183 (12.7) [10.5-15.2]

Abbreviations: FPL, federal poverty level; HMO, health maintenance organization.

^a^ Other includes American Indian or Alaska Native, Asian Indian, Chinese, Filipino, Japanese, Korean, Vietnamese, Other Asian, Native Hawaiian, Guamanian or Chamorro, Samoan, and Other Pacific Islander.

^b^ Other includes employer or company clinic, other hospital location including emergency department, urgent care center or walk-in facility, in-store health clinic, and some other place.

Adolescents reported the lowest frequencies of rating their contraceptive counseling clinician as excellent on at least 1 of the 4 PCCC items (45.3%; 95% CI, 37.8%-53.0% to 53.0%; 95% CI, 46.2%-59.6%), followed by young adults (53.2%; 95% CI, 45.1%-61.2% to 59.5%; 95% CI, 50.7%-67.7%) and adults (56.1%; 95% CI, 52.1%-60.0% to 64.2%; 95% CI, 60.3%-67.9%) (Table 2). One-third (35.8%; 95% CI, 29.0-43.2) of adolescents, 43.4% (95% CI, 36.9-50.2) of young adults, and 49.7% (95% CI, 45.9-53.5) of adults reported receiving overall PCCC (excellent on all 4 items). Across the 4 scale items, there were significant differences in the distribution of responses across age groups for the items “How did this provider rate on respecting you as a person?” and “How did this provider rate with respect to letting you say what mattered most to you about your birth control method?” (Table 2).

**Table 2.** Respondents’ Ratings of Their Family Planning Practitioner on the Person-Centered Contraceptive Counseling (PCCC) Scale, National Survey of Family Growth, 2022-2023

PCCC	Respondent age group, No. (%) [95% CI]		
	15-19 y (n = 261)	20-24 y (n = 276)	≥25 y (n = 1506)
**Overall PCCC**			
Excellent across all 4 items	97 (35.8) [29.0-43.2]	122 (43.4) [36.9-50.2]	765 (49.7) [45.9-53.5]
Less than excellent across all 4 items	164 (64.2) [56.8-71.0]	154 (56.6) [49.9-63.2]	741 (50.3) [46.5-54.1]
**Respecting you as a person**			
Excellent	132 (50.6) [42.9-58.3]	150 (53.2) [45.1-61.2]	940 (64.2) [60.3-67.9]
Very good	71 (28.5) [21.9-36.2]	53 (23.6) [16.7-32.3]	314 (19.2) [16.9-21.9]
Good	49 (18.0) [12.8-24.6]	58 (18.0) [12.8-24.7]	189 (12.5) [10.3-15.0]
Fair	9 (2.9) [1.3-6.6]	14 (5.0) [2.5-9.5]	51 (3.1) [2.1-4.5]
Poor	0	1 (0.2) [0.03-1.7]	12 (1.0) [0.4-2.4]
**Letting you say what mattered most to you about your birth control method**			
Excellent	126 (46.0) [39.8-52.3]	146 (56.4) [47.9-64.5]	894 (59.7) [55.6-63.7]
Very good	66 (27.7) [21.7-34.6]	58 (21.4) [15.1-29.3]	316 (20.3) [17.8-23.1]
Good	48 (17.2) [12.3-23.7]	49 (13.4) [9.3-18.9]	216 (15.0) [12.5-18.1]
Fair	19 (7.5) [4.3-12.8]	19 (7.8) [4.6-12.9]	63 (3.8) [2.7-5.3]
Poor	2 (1.6) [0.3-7.3]	4 (1.1) [0.3-4.8]	17 (1.2) [0.6-2.4]
**Taking your preferences about birth control seriously**			
Excellent	137 (53.0) [46.2-59.6]	156 (59.5) [50.7-67.7]	912 (60.3) [56.1-64.3]
Very good	57 (21.7) [16.8-27.6]	53 (19.8) [13.7-27.8]	309 (20.5) [17.6-23.7]
Good	52 (20.6) [14.9-27.8]	47 (14.1) [9.7-20.1]	204 (14.4) [12.0-17.3]
Fair	10 (2.8) [1.3-5.6]	16 (5.3) [2.8-9.8]	56 (2.9) [2.2-4.0]
Poor	5 (2.0) [0.6-6.8]	4 (1.3) [0.3-5.0]	25 (1.9) [1.1-3.4]
**Giving you enough information to make the best decision about your birth control method**			
Excellent	127 (45.3) [37.8-53.0]	145 (55.5) [46.8-63.9]	855 (56.1) [52.1-60.0]
Very good	65 (28.6) [21.7-36.8]	51 (17.6) [12.1-24.8]	321 (20.9) [18.3-23.9]
Good	46 (15.8) [11.2-21.9]	57 (19.2) [14.1-25.6]	230 (16.1) [13.6-19.1]
Fair	16 (6.3) [4.0-10.0]	19 (6.5) [3.6-11.3]	68 (4.4) [3.0-6.4]
Poor	7 (3.9) [1.5-9.5]	4 (1.3) [0.3-4.6]	32 (2.4) [1.5-3.7]

Table 3 shows results from multivariable logistic regression models regressing PCCC ratings on respondent demographics. Compared with adults, adolescents had significantly lower odds of receiving overall PCCC (aOR, 0.61; 99% CI, 0.38-0.99) (Table 3). They also had significantly lower odds of rating their clinician as excellent on the items “How did this provider rate on respecting you as a person?” and “How did this provider rate on letting you say what mattered most to you about your birth control method?” (Table 3). There were no significant differences in the receipt of overall PCCC or on individual items between young adults and adults (Table 3).

**Table 3.** Sociodemographic Characteristics Associated With Overall Person-Centered Contraceptive Counseling Rating and Reporting Excellent on Individual Items, Odds Ratios (ORs) From Multivariable Logistic Regression Models, National Survey of Family Growth, 2022-2023 (N = 2043)

Characteristic	Overall PCCC		Respecting you as a person		Letting you say what mattered most to you about your birth control method		Taking your preferences about birth control seriously		Giving you enough information to make the best decision about your birth control method	
	Unadjusted OR (99% CI)	aOR (99% CI)	Unadjusted OR (99% CI)	aOR (99% CI)	Unadjusted OR (99% CI)	aOR (99% CI)	Unadjusted OR (99% CI)	aOR (99% CI)	Unadjusted OR (99% CI)	aOR (99% CI)
Age, y										
15-19	0.56 (0.35-0.91)	0.61 (0.38-0.99)	0.57 (0.36-0.90)	0.61 (0.38-0.99)	0.57 (0.39-0.84)	0.61 (0.42-0.90)	0.74 (0.49-1.12)	0.82 (0.54-1.24)	0.65 (0.40-1.04)	0.70 (0.44-1.12)
20-24	0.78 (0.53-1.14)	0.83 (0.55-1.24)	0.63 (0.41-0.98)	0.65 (0.42-1.00)	0.87 (0.53-1.42)	0.91 (0.56-1.48)	0.97 (0.58-1.61)	1.04 (0.62-1.75)	0.98 (0.59-1.61)	1.05 (0.63-1.74)
≥25	1 [Reference]	1 [Reference]	1 [Reference]	1 [Reference]	1 [Reference]	1 [Reference]	1 [Reference]	1 [Reference]	1 [Reference]	1 [Reference]
Race and ethnicity										
Black	NA	0.69 (0.47-1.02)	NA	0.78 (0.50-1.21)	NA	0.74 (0.49-1.11)	NA	0.68 (0.45-1.05)	NA	0.77 (0.52-1.13)
Hispanic	NA	1.23 (0.89-1.69)	NA	1.22 (0.87-1.70)	NA	1.22 (0.86-1.74)	NA	1.23 (0.89-1.70)	NA	1.12 (0.80-1.56)
Other^a^	NA	0.81 (0.52-1.27)	NA	0.93 (0.62-1.39)	NA	0.75 (0.47-1.19)	NA	0.72 (0.48-1.08)	NA	0.90 (0.61-1.34)
White	NA	1.13 (0.86-1.48)	NA	1.02 (0.75-1.37)	NA	1.14 (0.88-1.48)	NA	1.19 (0.91-1.57)	NA	1.10 (0.85-1.41)
English proficiency										
Low	NA	0.36 (0.19-0.69)	NA	0.37 (0.21-0.65)	NA	0.37 (0.21-0.65)	NA	0.39 (0.21-0.72)	NA	0.40 (0.24-0.68)
High	NA	1 [Reference]	NA	1 [Reference]	NA	1 [Reference]	NA	1 [Reference]	NA	1 [Reference]
Income level, % FPL										
>100	NA	1.54 (0.91-2.61)	NA	1.62 (1.02-2.59)	NA	1.64 (0.99-2.72)	NA	1.70 (1.08-2.68)	NA	1.53 (0.89-2.64)
≤100	NA	1 [Reference]	NA	1 [Reference]	NA	1 [Reference]	NA	1 [Reference]	NA	1 [Reference]
Where birth control services were received										
Public clinic	NA	0.56 (0.37-0.85)	NA	0.57 (0.36-0.88)	NA	0.65 (0.41-1.03)	NA	0.58 (0.37-0.91)	NA	0.64 (0.41-0.99)
Private doctor's office or HMO facility	NA	1 [Reference]	NA	1 [Reference]	NA	1 [Reference]	NA	1 [Reference]	NA	1 [Reference]
Other^b^	NA	0.43 (0.24-0.75)	NA	0.55 (0.35-0.88)	NA	0.51 (0.30-0.89)	NA	0.41 (0.24-0.71)	NA	0.38 (0.22-0.65)

Abbreviations: aOR, adjusted odds ratio; FPL, federal poverty level; HMO, health maintenance organization; NA, not available; OR, odds ratio.

^a^ Other includes American Indian or Alaska Native, Asian Indian, Chinese, Filipino, Japanese, Korean, Vietnamese, Other Asian, Native Hawaiian, Guamanian or Chamorro, Samoan, and Other Pacific Islander.

^b^ Other includes employer or company clinic, other hospital location including emergency department, urgent care center or walk-in facility, in-store health clinic, and some other place.

Respondents with low English proficiency (aOR, 0.36; 99% CI, 0.19-0.69) and respondents who received their birth control services at a public clinic (aOR, 0.56; 99% CI, 0.37-0.85) or another type of facility (aOR, 0.43; 99% CI, 0.24-0.75) also had significantly lower odds of rating their clinician as excellent on all items (and overall PCCC) than respondents with high English proficiency and those who received their services at a private doctor's office or HMO facility. Conversely, respondents with incomes above the FPL had significantly higher odds of rating their clinician as excellent on the items “How did this provider rate on respecting you as a person?” (aOR, 1.62; 99% CI, 1.02-2.59) and “How did this provider rate on taking your preferences about birth control seriously?” (aOR, 1.70; 99% CI, 1.08-2.68) than respondents at or below the FPL.

Table 4 presents results from multivariable logistic regression models that regress preferred contraceptive method use on receipt of overall PCCC by age group. Both bivariate and multivariable models show that adolescents who received overall PCCC had significantly higher odds of using their preferred contraceptive method than adolescents who did not receive PCCC (unadjusted odds ratio, 2.26; 95% CI, 1.02-5.00]; aOR, 2.35; 95% CI, 1.06-5.21). Race and ethnicity, English proficiency, income level, and where birth control services were received were not significantly associated with preferred contraceptive method use among adolescents. Conversely, there were no significant differences in preferred contraceptive method use by PCCC among young adults (aOR, 0.57; 95% CI, 0.25-1.26) or adults (aOR, 1.01; 95% CI, 0.70-1.45) (Table 4).

**Table 4.** Association Between Overall Person-Centered Contraceptive Counseling (PCCC) and Use of Preferred Contraceptive Method Among Adolescents, Odds Ratios From Multivariable Logistic Regression Models, National Survey of Family Growth, 2022-2023

Characteristic	15-19 y (n = 213)		20-24 y (n = 260)		≥25y (n = 1452)	
	Unadjusted OR (95% CI)	aOR (95% CI)	Unadjusted OR (95% CI)	aOR (95% CI)	Unadjusted OR (95% CI)	aOR (95% CI)
PCCC						
Yes	2.26 (1.02-5.00)	2.35 (1.06-5.21)	0.77 (0.39-1.54)	0.57 (0.25-1.26)	1.16 (0.81-1.65)	1.01 (0.70-1.45)
No	1 [Reference]	1 [Reference]	1 [Reference]	1 [Reference]	1 [Reference]	1 [Reference]
Race and ethnicity						
Black	NA	0.64 (0.20-2.08)	NA	0.70 (0.26-1.92)	NA	1.08 (0.66-1.78)
Hispanic	NA	1.07 (0.37-3.14)	NA	0.57 (0.26-1.21)	NA	1.00 (0.65-1.54)
Other^a^	NA	0.93 (0.27-3.22)	NA	0.40 (0.11-1.51)	NA	1.09 (0.70-1.70)
White	NA	1.21 (0.47-3.11)	NA	2.60 (1.10-6.13)	NA	0.93 (0.64-1.35)
English proficiency						
Low	NA	3.38 (0.68-16.72)	NA	0.20 (0.07-0.56)	NA	0.62 (0.42-0.92)
High	NA	1 [Reference]	NA	1 [Reference]	NA	1 [Reference]
Income level, % FPL						
>100	NA	1.15 (0.48-2.77)	NA	1.80 (0.74-4.36)	NA	1.26 (0.81-1.94)
≤100	NA	1 [Reference]	NA	1 [Reference]	NA	1 [Reference]
Where birth control services were received						
Public clinic	NA	1.27 (0.44-3.67)	NA	0.92 (0.41-2.07)	NA	0.44 (0.29-0.67)
Private doctor's office or HMO facility	NA	1 [Reference]	NA	1 [Reference]	NA	1 [Reference]
Other^b^	NA	0.73 (0.32-1.69)	NA	0.71 (0.23-2.19)	NA	0.88 (0.47-1.65)

Abbreviations: aOR, adjusted odds ratio; FPL, federal poverty level; NA, not available; OR, odds ratio.

^a^ Other includes American Indian or Alaska Native, Asian Indian, Chinese, Filipino, Japanese, Korean, Vietnamese, Other Asian, Native Hawaiian, Guamanian or Chamorro, Samoan, and Other Pacific Islander.

^b^ Other includes employer or company clinic, other hospital location including emergency department, urgent care center or walk-in facility, in-store health clinic, and some other place.

### Sensitivity Analysis

Due to changes in interview mode and notable declines in response rates in the 2022 to 2023 NSFG wave, the National Center for Health Statistics has advised against making direct comparisons between the 2022 to 2023 wave and previous cycles of the survey.^33^ Accordingly, we did not pool data across waves or make formal statistical comparisons of estimates over time. However, to assess the robustness of our findings and ensure that observed associations were not unique to the 2022 to 2023 sample, we replicated our analyses using the 2017 to 2019 NSFG data. These analyses are not intended for cross-sample comparison, but rather to assess whether the direction and magnitude of associations were similar. Findings related to overall receipt of PCCC across adolescent, young adult, and older adult age groups were consistent in both waves, although some variation in responses to individual PCCC items were observed. Results regarding the relationship between receipt of PCCC and use of a preferred contraceptive method among age groups were also similar across samples. Results for the 2017 to 2019 sample are in eTables 2 to 5 in Supplement 1.

## Discussion

This study found that receipt of PCCC was low across all age groups, with only one-third to one-half reporting receiving PCCC at their last family planning visit. However, adolescents aged 15 to 19 years had significantly lower odds of receiving PCCC than adults aged 25 years or older, both in the aggregate measure and across 2 of the 4 individual components of PCCC. Notably, adolescents were significantly less likely to rate their clinicians as excellent on items about respecting them as a person and letting them say what mattered most to them about birth control. These findings indicated that while receipt of PCCC was suboptimal across all age groups, notable disparities existed for young people—particularly adolescents—who were least likely to receive high-quality, person-centered contraceptive care.

These findings aligned with previous research indicating that nearly one-third of adolescents and young adults felt they lacked sufficient information to make a decision about the best contraceptive method for them.^34^ When adolescents and young adults experienced information gaps or negative encounters in clinical settings, they frequently turned to alternative sources, particularly social media,^35^ potentially exposing them to misinformation and disinformation about contraception.^36,37^ This was particularly concerning as research has shown that adolescents and young adults may modify their behavior in response to information received online.^38,39^ Despite turning to online sources for contraceptive information, research showed that adolescents and young adults would prefer to receive this information from a health care professional.^40^ This underscored the importance of ensuring adolescents and young adults receive quality contraceptive counseling from their clinicians and can access that care.

The second key finding from this study was that while adolescents were less likely to receive PCCC than adults, when they did receive such counseling, it was associated with increased likelihood of using their preferred contraceptive method. This supported findings from recent NSFG research, which found that low-income contraceptive users who received PCCC had lower odds of unfulfilled contraceptive preferences due to cost.^25^ Interestingly, this study did not find differences in preferred contraceptive method use by PCCC among young adults or adults, differing from some prior research.^15^ This may be attributed to the NSFG’s measure of preferred contraceptive use, which asked whether respondents would use a different method if cost were not a factor. For adolescents, receiving PCCC may have played a more influential role in helping them navigate options within their financial constraints and identified affordable methods that aligned more closely with their preferences—something adults may be better equipped to do independently due to greater experience, resources, or autonomy.

Ensuring that clinicians serving adolescents are trained to provide person-centered contraceptive counseling should be prioritized. Clinical settings should also consider ways to obtain adolescent patient feedback to ensure their contraceptive counseling preferences are being met. Additionally, efforts should be made to ensure clinical settings are adolescent-friendly,^41^ as services that are tailored to meet the needs of young people may improve reproductive health outcomes.^42^

### Limitations

This study has limitations. First, the measure of preferred contraceptive method use in the NSFG is not ideal for capturing other domains of method satisfaction beyond cost. As a result, it overlooks other important dimensions of method satisfaction and dissatisfaction, such as adverse effects, access, confidentiality, or personal preferences.^43^ Other studies of adolescents should include more robust measures of preferred contraceptive method use.

Second, this study could not examine variation by clinician type (eg, obstetrics/gynecology, family medicine, pediatrician, midwife, advanced practice clinician, etc), which could be an important mediating factor in the quality of PCCC that adolescents receive and warrants investigation in future research. Third, the NSFG does not ask directly about gender identity, and some respondents may identify in the survey differently from their biological sex at birth.

Lastly, it is worth questioning whether PCCC as currently conceptualized is the most appropriate measure for evaluating contraceptive counseling quality for adolescents. The current PCCC measure has not been validated with robust adolescent populations and notably does not address clinician coercion or other forms of pressure and influence on contraceptive choices—an issue to which adolescents are particularly vulnerable.^44,45^ Future research should validate the PCCC scale for adolescent populations or develop a psychometrically validated measure designed specifically for this population, given their unique needs.

Despite these limitations, our findings underscore the importance of implementing strategies to improve PCCC for adolescents, given its positive association with preferred contraceptive method use.^46^ Health care professionals who work with adolescents should receive specialized training in delivering age-appropriate contraceptive counseling that emphasizes information sharing and creates space for adolescents to express their preferences.

## Conclusions

In this survey study of 2043 female-identifying respondents who had a family planning visit in the past year, we found that receipt of PCCC was associated with higher rates of preferred contraceptive method use for adolescents, while highlighting gaps in adolescents’ receipt of this care. Although adolescents were less likely to receive PCCC, when they did receive PCCC, they reported higher rates of preferred method use. These findings support ongoing efforts to improve the quality of contraceptive counseling for adolescents through person-centered approaches that support their autonomy, provide comprehensive information, and respect their preferences. Policy makers, health care systems, and individual clinicians should prioritize the development and implementation of adolescent-specific PCCC strategies to ensure that adolescents have access to high-quality contraceptive care that meets their unique needs.

## Notes

Supplement 1. **eTable 1.** Full sample characteristics, female-identifying respondents National Survey of Family Growth, 2022-2023 **eTable 2. **Sample characteristics, female-identifying respondents who received a birth control method and/or counseling in the past year, National Survey of Family Growth, 2017-2019 **eTable 3.** Respondents’ ratings of their family planning provider on the Person-Centered Contraceptive Counseling Scale, National Survey of Family Growth, 2017-2019 **eTable 4.** Sociodemographic characteristics associated with overall Person-Centered Contraceptive Counseling rating and individual items, odds ratios from multivariate logistic regression models, National Survey of Family Growth, 2017-2019 **eTable 5.** Association between overall Person-Centered Contraceptive Counseling and use of preferred contraceptive method among adolescents, odds ratios from multivariate logistic regression models, National Survey of Family Growth, 2017-2019

Supplement 2.
Data Sharing Statement
