# Supplementary material for: Adolescents’ and Young Adults’ Receipt of Person-Centered Contraceptive Counseling
Source: JAMA Netw Open. 2025 Dec 26;8(12):e2551287. doi: 10.1001/jamanetworkopen.2025.51287 (PMC12743274; doi:10.1001/jamanetworkopen.2025.51287)
Supplement: Supplement 1. — eTable 1. Full sample characteristics, female-identifying respondents National Survey of Family Growth, 2022-2023 eTable 2. Sample characteristics, female-identifying respondents who received a birth control method and/or counseling in the past year, National Survey of Family Growth, 2017-2019 eTable 3. Respondents’ ratings of their family planning provider on the Person-Centered Contraceptive Counseling Scale, National Survey of Family Growth, 2017-2019 eTable 4. Sociodemographic characteristics associated with overall Person-Centered Contraceptive Counseling rating and individual items, odds ratios from multivariate logistic regression models, National Survey of Family Growth, 2017-2019 eTable 5. Association between overall Person-Centered Contraceptive Counseling and use of preferred contraceptive method among adolescents, odds ratios from multivariate logistic regression models, National Survey of Family Growth, 2017-2019 [file jamanetwopen-e2551287-s001.pdf]

Supplemental Online Content

Whitfield B, Wilkinson TA, Lindberg LD, et al. Adolescents' and young adults' receipt of person-centered contraceptive counseling. *JAMA Netw Open*. 2025;8(12):e2551287. doi:10.1001/jamanetworkopen.2025.51287

eTable 1. Full sample characteristics, female-identifying respondents National Survey of Family Growth, 2022-2023

eTable 2. Sample characteristics, female-identifying respondents who received a birth control method and/or counseling in the past year, National Survey of Family Growth, 2017-2019

eTable 3. Respondents' ratings of their family planning provider on the Person-Centered Contraceptive Counseling Scale, National Survey of Family Growth, 2017-2019

eTable 4. Sociodemographic characteristics associated with overall Person-Centered Contraceptive Counseling rating and individual items, odds ratios from multivariate logistic regression models, National Survey of Family Growth, 2017-2019

eTable 5. Association between overall Person-Centered Contraceptive Counseling and use of preferred contraceptive method among adolescents, odds ratios from multivariate logistic regression models, National Survey of Family Growth, 2017-2019

This supplemental material has been provided by the authors to give readers additional information about their work.

© 2025 Whitfield B et al. *JAMA Network Open*.

eTable 1. Full sample characteristics, female-identifying respondents National Survey of Family Growth, 2022–2023

Characteristics	Total (N=5,586) No. (%) 95% CI	15-19 (N=730) No. (%) 95% CI	20-24 (N=606) No. (%) 95% CI	25+ (N=4,250) No. (%) 95% CI
Race, ethnicity, and English proficiency				
Black	844 (13.1) [10.79-15.78]	110 (12.8) [9.66-16.85]	98 (13.4) [10.04-17.77]	636 (13.1) [10.68-15.88]
Hispanic	1,232 (21.6) [17.86-25.94]	218 (25.2) [20.00-31.17]	173 (23.6) [18.22-29.94]	841 (20.5) [16.90-24.72]
Other[a]	721 (12.7) [10.25-15.62]	87 (12.6) [9.93-15.81]	81 (11.6) [8.49-15.75]	553 (12.9) [10.22-16.23]
White	2,789 (52.6) [47.90-57.24]	315 (49.4) [43.35-55.50]	254 (51.3) [44.54-58.10]	2,220 (53.5) [48.72-58.16]
English proficiency				
Low	660 (12.6) [11.02-14.45]	68 (8.4) [5.96-11.66]	55 (7.9) [5.76-10.82]	537 (14.4) [12.46-16.63]
High	4,887 (87.4) [85.55-88.98]	657 (91.6) [88.34-94.04]	547 (92.1) [89.18-94.24]	3,683 (85.6) [83.37-87.54]
Income level				
0-100% FPL	1,085 (18.8) [16.88-20.96]	189 (24.3) 20.90-28.07]	145 (19.8) [15.95-24.40]	751 (17.6) [15.52-19.81]
101-250% FPL	1,478 (26.3) [23.95-28.69]	229 (29.5) [25.26-34.10]	200 (31.5) [26.88-36.46]	1,049 (24.6) [22.11-27.17]
>250% FPL	3,023 (54.9) [51.18-58.58]	312 (46.2) [40.90-51.60]	261 (48.7) [42.46-54.96]	2,450 (57.9) [54.18-61.50]
Where birth control services were received				
Private doctor's office or HMO facility	1,366 (65.9) [62.60-68.97]	137 (52.8) [44.30-61.08]	161 (58.3) [51.49-67.74]	1,068 (70.8) [66.99-74.35]
Clinic	382 (19.0) [16.31-21.96]	65 (27.6) [20.35-36.28]	68 (22.5) [16.32-30.10]	249 (16.1) [13.35-19.37]
Other[b]	303 (15.2) [13.31-17.24]	62 (19.6) [14.47-26.05]	52 (19.3) [14.27-25.50]	189 (13.1) [10.79-15.73]

[a]Other includes American Indian or Alaska Native, Asian Indian, Chinese, Filipino, Japanese, Korean, Vietnamese, Other Asian, Native Hawaiian, Guamanian or Chamorro, Samoan, Other Pacific Islander
[b]Other includes employer or company clinic, other hospital location including emergency room, urgent care center or walk-in facility, in-store health clinic (like CVS, Target, or Walmart), and some other place

© 2025 Whitfield B et al. *JAMA Network Open*.

eTable 2. Sample characteristics, female-identifying respondents who received a birth control method and/or counseling in the past year, National Survey of Family Growth, 2017–2019

	Total (N=2,228) No. (%) 95% CI	15-19 (N=360) No. (%) 95% CI	20-24 (N=441) No. (%) 95% CI	25+ (N=1,427) No. (%) 95% CI
Race, ethnicity, and English proficiency				
Black	439 (13.8) [10.62-17.64]	69 (14.1) [9.26-21.02]	79 (12.7) [9.08-17.59]	291 (14.0) [10.36-18.64]
Hispanic	565 (18.4) [14.35-23.37]	88 (14.1) [9.67-20.00]	121 (19.3) [13.93-26.00]	356 (19.2) [14.63-24.68]
Other[a]	190 (10.0) [7.56-13.01]	22 (7.3) [3.90-13.34]	40 (11.2) [6.51-18.68]	128 (10.1) [7.75-13.16]
White	1,034 (57.8) [52.87-62.67]	181 (64.5) [56.23-71.94]	201 (56.8) [50.50-62.84]	652 (56.7) [50.54-62.66]
English proficiency				
Low	243 (9.0) [7.14-11.37]	27 (7.5) [4.05-13.55]	36 (4.9) [3.24-7.41]	180 (10.7) [8.11-14.03]
High	1,979 (91.0) [88.63-92.86]	333 (92.5) [86.45-95.95]	405 (95.1) [92.59-96.76]	1,241 (89.3) [85.97-91.89]
Income level				
0-100% FPL	550 (20.8) [18.44-23.33]	124 (32.9) [26.67-39.89]	110 (22.8) [16.82-30.21]	316 (17.4) [14.99-20.03]
101-250% FPL	691 (28.2) [25.37-31.21]	118 (32.4) [26.14-39.40]	168 (38.5) [32.46-45.01]	405 (23.9) [20.89-27.20]
>250% FPL	987 (51.0) [47.44-54.59]	118 (34.6) [27.34-42.73]	163 (38.6) [31.51-46.26]	706 (58.7) [54.71-62.64]
Where birth control services were received				
Private doctor's office or HMO facility	1,537 (73.0) [69.07-76.52]	229 (63.2) 54.99-70.67]	281 (68.0) [59.12-75.72]	1,027 (76.8) [73.16-80.01]
Clinic	513 (19.3) [16.53-22.35]	101 (27.7) [20.38-36.36]	118 (21.4) [16.38-27.44]	294 (16.7) [14.05-19.72]
Other[b]	175 (7.8) [5.74-10.45]	28 (9.2) [5.54-14.78]	41 (10.6) [5.71-18.92]	106 (6.5) [4.96-8.60]

[a]Other includes American Indian or Alaska Native, Asian Indian, Chinese, Filipino, Japanese, Korean, Vietnamese, Other Asian, Native Hawaiian, Guamanian or Chamorro, Samoan, Other Pacific Islander

[b]Other includes employer or company clinic, other hospital location including emergency room, urgent care center or walk-in facility, in-store health clinic (like CVS, Target, or Walmart), and some other place

© 2025 Whitfield B et al. *JAMA Network Open*.

eTable 3. Respondents' ratings of their family planning provider on the Person-Centered Contraceptive Counseling Scale, National Survey of Family Growth, 2017-2019

Person-Centered Contraceptive Counseling Measure	15-19 (N=360) No. (%) 95% CI	20-24 (N=441) No. (%) 95% CI	25+ (N=1,427) No. (%) 95% CI
Overall PCCC			
Less than excellent across all 4 measures	214 (58.8) [51.45-65.73]	244 (53.8) [45.32-61.99]	687 (45.6) [41.36-49.89]
Excellent across all 4 measures	146 (41.2) [34.27-48.55]	197 (46.2) [38.01-54.68]	740 (54.4) [50.11-58.64]
Respecting you as a person			
Poor	3 (0.5) [0.15-1.53]	2 (0.2) [0.04-0.89]	5 (0.1) [0.05-0.37]
Fair	14 (3.2) [1.63-6.17]	12 (3.2) [1.09-8.98]	25 (1.6) [0.89-2.91]
Good	33 (10.8) [6.61-17.22]	45 (9.2) [6.00-14.00]	156 (9.7) [7.34-12.62]
Very good	86 (23.5) [17.34-31.00]	88 (18.8) [14.50-23.98]	285 (18.1) [15.11-21.56]
Excellent	224 (62.0) [53.23-70.08]	294 (68.6) [62.37-74.19]	956 (70.5) [66.06-74.53]
Letting you say what mattered most to you about your birth control method			
Poor	9 (1.3) [0.62-2.56]	3 (0.4) [0.11-1.25]	11 (0.6) [0.29-1.32]
Fair	9 (2.2) [1.05-4.44]	16 (3.2) [1.52-6.66]	40 (2.5) [1.48-4.33]
Good	38 (12.8) [7.711-20.42]	61 (13.4) [9.78-18.21]	158 (10.6) [8.38-13.32]
Very good	96 (26.7) [20.95-33.38]	89 (20.7) [14.80-28.25]	286 (17.9) [15.35-20.71]
Excellent	208 (57.1) [49.27-64.57]	272 (62.2) [53.72-70.07]	932 (68.4) [64.20-72.27]
Taking your preferences about birth control seriously			
Poor	6 (1.1) [0.43-2.71]	4 (0.7) [0.18-2.44]	16 (0.7) [0.41-1.34]
Fair	14 (3.4) [1.59-7.24]	17 (3.2) [1.81-5.55]	33 (2.2) [1.25-3.82]
Good	38 (11.5) [7.56-17.00]	41 (10.8) [6.90-16.41]	154 (10.8) [8.42-13.74]
Very good	76 (22.8) [16.68-30.46]	98 (22.4) [16.01-30.53]	268 (15.6) [13.33-18.08]
Excellent	226 (61.2) [53.59-68.23]	281 (62.9) [54.98-70.26]	956 (70.7) [66.55-74.56]

© 2025 Whitfield B et al. *JAMA Network Open*.

Person-Centered Contraceptive Counseling Measure	15-19 (N=360)<br>No. (%)<br>95% CI	20-24 (N=441)<br>No. (%)<br>95% CI	25+ (N=1,427)<br>No. (%)<br>95% CI
Giving you enough information to make the best decision about your birth control method			
Poor	5 (0.9)<br>[0.35-2.39]	5 (1.0)<br>0.41-2.58]	23 (1.4)<br>[0.71-2.81]
Fair	19 (6.7)<br>[3.59-12.03]	22 (7.5)<br>[4.00-13.78]	46 (2.8)<br>[1.79-4.25]
Good	45 (12.6)<br>[8.87-17.62]	50 (11.2)<br>[7.26-16.96]	194 (14.0)<br>[11.38-17.06]
Very good	96 (23.4)<br>[18.16-29.61]	123 (26.3)<br>[18.60-35.85]	309 (20.1)<br>[17.24-23.33]
Excellent	195 (56.4)<br>[48.73-63.81]	241 (53.9)<br>[44.97-62.52]	855 (61.7)<br>[57.59-65.70]

© 2025 Whitfield B et al. *JAMA Network Open*.

eTable 4. Sociodemographic characteristics associated with overall Person-Centered Contraceptive Counseling rating and individual items, odds ratios from multivariate logistic regression models, National Survey of Family Growth, 2017-2019 (N=2,228)

	Overall PCCC		Respecting you as a person		Letting you say what mattered most to you about your birth control method		Taking your preferences about birth control seriously		Giving you enough information to make the best decision about your birth control method	
Characteristics	Unadjusted OR [99% CI]	aOR [99% CI]	Unadjusted OR [99% CI]	aOR [99% CI]	Unadjusted OR [99% CI]	aOR [99% CI]	Unadjusted OR [99% CI]	aOR [99% CI]	Unadjusted OR [99% CI]	aOR [99% CI]
Age (ref=25+)										
15-19	0.59	0.62	0.68	0.73	0.62	0.63	0.65	0.69	0.80	0.82
	[0.37-0.95]	[0.39-0.98]	[0.42-1.11]	[0.45-1.18]	[0.39-0.98]	[0.40-0.99]	[0.40-1.06]	[0.42-1.13]	[0.50-1.28]	[0.52-1.32]
20-24	0.72	0.71	0.91	0.91	0.76	0.75	0.70	0.69	0.72	0.71
	[0.45-1.15]	[0.45-1.13]	[0.60-1.40]	[0.60-1.38]	[0.48-1.21]	[0.47-1.17]	[0.43-1.16]	[0.42-1.13]	[0.45-1.17]	[0.44-1.13]
Race and ethnicity										
Black		0.82		0.67		0.91		0.83		0.87
		[0.62-1.26]		[0.48-0.94]		[0.69-1.20]		[0.62-1.11]		[0.65-1.15]
Hispanic		0.96		1.14		0.81		0.92		0.94
		[0.68-1.36]		[0.77-1.67]		[0.57-1.15]		[0.65-1.30]		[0.68-1.30]
Other[a]		0.84		0.81		0.84		1.05		0.73
		[0.56-1.26]		[0.52-1.29]		[0.56-1.27]		[0.71-1.55]		[0.49-1.07]
White		1.20		1.20		1.28		1.14		1.26
		[0.94-1.54]		[0.89-1.62]		[0.98-1.67]		[0.88-1.48]		[0.96-1.64]
English proficiency (ref=High)										
Low		0.62		0.54		0.52		0.51		0.72
		[0.33-1.16]		[0.28-1.01]		[0.30-0.89]		[0.26-0.98]		[0.39-1.33]
Income level (ref=<100% FPL)										
>100% FPL		1.62		1.64		1.65		1.78		1.51
		[1.10-2.37]		[1.12-2.41]		[1.09-2.50]		[1.10-2.87]		[1.00-2.29]

© 2025 Whitfield B et al. *JAMA Network Open*.

Characteristics	Overall PCCC		Respecting you as a person		Letting you say what mattered most to you about your birth control method		Taking your preferences about birth control seriously		Giving you enough information to make the best decision about your birth control method	
	Unadjusted OR [95% CI]	aOR [95% CI]	Unadjusted OR [95% CI]	aOR [95% CI]	Unadjusted OR [95% CI]	aOR [95% CI]	Unadjusted OR [95% CI]	aOR [95% CI]	Unadjusted OR [95% CI]	aOR [95% CI]
Where birth control services were received (ref=Private doctor's office or HMO facility)										
Clinic		0.89		0.83		0.93		0.90		0.97
		[0.56-1.43]		[0.50-1.36]		[0.54-1.61]		[0.51-1.58]		[0.61-1.55]
Other[b]		0.90		0.71		0.80		0.75		1.30
		[0.41-1.97]		[0.33-1.50]		[0.37-1.75]		[0.37-1.52]		[0.66-2.54]

[a]Other includes American Indian or Alaska Native, Asian Indian, Chinese, Filipino, Japanese, Korean, Vietnamese, Other Asian, Native Hawaiian, Guamanian or Chamorro, Samoan, Other Pacific Islander

[b]Other includes employer or company clinic, other hospital location including emergency room, urgent care center or walk-in facility, in-store health clinic (like CVS, Target, or Walmart), and some other place

© 2025 Whitfield B et al. *JAMA Network Open*.

eTable 5. Association between overall Person-Centered Contraceptive Counseling and use of preferred contraceptive method among adolescents, odds ratios from multivariate logistic regression models, National Survey of Family Growth, 2017-2019

	15-19 (N=332)		20-24 (N=430)		25+ (N=1,403)	
Person-Centered Contraceptive Measure	Unadjusted OR [95% CI]	aOR [95% CI]	Unadjusted OR [95% CI]	aOR [95% CI]	Unadjusted OR [95% CI]	aOR [95% CI]
PCCC (ref=No)						
Yes	3.38	3.07	1.11	0.97	1.41	1.31
	[1.68-6.78]	[1.55-6.06]	[0.50-2.47]	[0.47-1.98]	[1.00-2.00]	[0.92-1.85]
Race and ethnicity						
Black		1.10		1.90		0.98
		[0.50-2.42]		[0.58-6.19]		[0.61-1.58]
Hispanic		0.70		1.33		0.67
		[0.33-1.47]		[0.63-2.83]		[0.45-0.99]
Other[a]		0.75		0.46		0.84
		[0.17-3.32]		[0.15-1.37]		[0.46-1.51]
White		1.35		0.81		1.41
		[0.67-2.72]		[0.37-1.79]		[0.95-2.09]
English proficiency (ref=High)						
Low		0.81		1.05		0.86
		[0.16-4.05]		[0.34-3.30]		[0.49-1.52]
Income level (ref=<100% FPL)						
>100% FPL		1.44		0.93		1.44
		[0.76-2.72]		[0.45-1.90]		[0.92-2.27]
Where birth control services were received (ref=Private doctor's office or HMO facility)						
Clinic		1.24		0.30		0.75
		[0.52-2.98]		[0.13-0.66]		[0.48-1.19]
Other[b]		0.53		0.16		0.56
		1.24		0.30		0.75

[a]Other includes American Indian or Alaska Native, Asian Indian, Chinese, Filipino, Japanese, Korean, Vietnamese, Other Asian, Native Hawaiian, Guamanian or Chamorro, Samoan, Other Pacific Islander
[b]Other includes employer or company clinic, other hospital location including emergency room, urgent care center or walk-in facility, in-store health clinic (like CVS, Target, or Walmart), and some other place

© 2025 Whitfield B et al. *JAMA Network Open*.
