# Supplementary material for: Adolescents’ and Young Adults’ Receipt of Person-Centered Contraceptive Counseling
Source: JAMA Netw Open. 2025 Dec 26;8(12):e2551287. doi: 10.1001/jamanetworkopen.2025.51287 (PMC12743274; doi:10.1001/jamanetworkopen.2025.51287)
Supplement: Supplement 2. — Data Sharing Statement [file jamanetwopen-e2551287-s002.pdf]

Data Sharing Statement

Data
Data available: Yes
Data types: Deidentified participant data
How to access data: They are publicly available through the National Survey of Family Growth
When available: With publication

Supporting Documents
Document types: Statistical/analytic code
How to access documents: They can be requested by emailing bwhitfield@utexas.edu
When available: With publication

Additional Information
Who can access the data: Anyone requesting
Types of analyses: Any purpose
Mechanisms of data availability: N/A they are publicly available
